# A cautionary note on the use of Ornstein Uhlenbeck models in macroevolutionary studies

**DOI:** 10.1111/bij.12701

**Published:** 2015-12-01

**Authors:** Natalie Cooper, Gavin H. Thomas, Chris Venditti, Andrew Meade, Rob P. Freckleton

**Affiliations:** ^1^ School of Natural SciencesTrinity College DublinDublin 2Ireland; ^2^ Trinity Centre for Biodiversity ResearchTrinity College DublinDublin 2Ireland; ^3^ Department of Life SciencesNatural History MuseumCromwell RoadLondonSW7 5BDUK; ^4^ Department of Animal and Plant SciencesUniversity of SheffieldSheffieldS10 2TNUK; ^5^ School of Biological SciencesUniversity of ReadingReadingBerkshireRG6 6BXUK

**Keywords:** comparative methods, macroevolutionary models, OU, phylogeny, stabilizing selection

## Abstract

Phylogenetic comparative methods are increasingly used to give new insights into the dynamics of trait evolution in deep time. For continuous traits the core of these methods is a suite of models that attempt to capture evolutionary patterns by extending the Brownian constant variance model. However, the properties of these models are often poorly understood, which can lead to the misinterpretation of results. Here we focus on one of these models – the Ornstein Uhlenbeck (OU) model. We show that the OU model is frequently incorrectly favoured over simpler models when using Likelihood ratio tests, and that many studies fitting this model use datasets that are small and prone to this problem. We also show that very small amounts of error in datasets can have profound effects on the inferences derived from OU models. Our results suggest that simulating fitted models and comparing with empirical results is critical when fitting OU and other extensions of the Brownian model. We conclude by making recommendations for best practice in fitting OU models in phylogenetic comparative analyses, and for interpreting the parameters of the OU model.

## Introduction

Phylogenetic comparative methods (PCMs) are powerful tools for identifying patterns in the evolution of species traits, and for potentially inferring the evolutionary processes that underlie them (e.g. Freckleton, 2009; Nunn, 2011; O'Meara, 2012; Pennell & Harmon, 2013). These approaches have been used, for example, to infer potential rates of species responses to climate change (Quintero & Wiens, 2013), test the role of ecological niche as a driver of morphological evolution (Pienaar *et al*., 2013) and test for constraints in adaptive radiations (Blackburn *et al*., 2013).

The majority of PCMs use an explicit evolutionary model to characterize trait evolution (Freckleton *et al*. 2011). Most model‐based methods for characterizing trait evolution are based on the Brownian constant variance model (for exceptions see Price, 1997; Harvey & Rambaut, 2000; Freckleton & Harvey, 2006). The Brownian model, first applied in a phylogenetic context by Cavalli‐Sforza & Edwards (1967) and to across‐species data by Felsenstein (1973), is a simple model of trait evolution in which trait variance accrues as a linear function of time, and makes the prediction that traits of closely related species are more similar than those of distantly related ones. The Brownian model has been modified in various ways to account for a suite of ecological and evolutionary processes (e.g. Grafen, 1989; Hansen, 1997; Pagel, 1997, 1999). Most of these involve a transformation of the tree and thereby fitting a model with one or more extra parameters. These modified Brownian models tend to fit better and often have links to process‐based interpretations.

One of the most commonly used Brownian‐like models is the Ornstein Uhlenbeck (OU) model. The OU model was introduced to population genetics by Lande (1976) to model stabilizing selection in which the trait is drawn towards a fitness optimum on an adaptive landscape. The process operating in comparative data is analogous to but distinct from stabilizing selection. The phylogenetic OU model is a modification of the Brownian model with an additional parameter α that measures the strength of return towards a theoretical optimum (Hansen, 1997) that is shared across a clade or subset of species. Although widely used, the properties of the OU model, and other direct extensions of the Brownian model, are poorly understood leading to the potential for inappropriate use and misinterpretation of results.

In this paper we present an introduction to the OU model, its general properties and some issues with its use in ecology, evolution and palaeontology. We use simulations to demonstrate the inherent bias in estimating the core parameter of the OU model, α, that describes the strength of pull towards a central value (typically referred to as the trait or selective optima). We discuss the intricacies of interpreting OU models biologically, and provide advice for appropriate use of OU models in phylogenetic comparative analyses. We also show that very small amounts of intraspecific trait variation (including measurement error) can profoundly affect the performance of models. These findings will be applicable to other models of evolution, but we focus on the OU model because of its widespread use and because of the ambiguity in the link between pattern and process when interpreting estimates of the α parameter. We are not the first to describe some of these problems (e.g. Ives & Garland, 2010; Boettiger, Coop & Ralph, 2012; Hansen & Bartoszek, 2012; Ho & Ané, 2013, 2014). However, widespread use of the model is clear evidence that many are unaware of the potential problems. We use a simulation approach to summarize the problems and to generate practical recommendations of how to deal with them.

### Uses of the OU model

**Figure 1:**
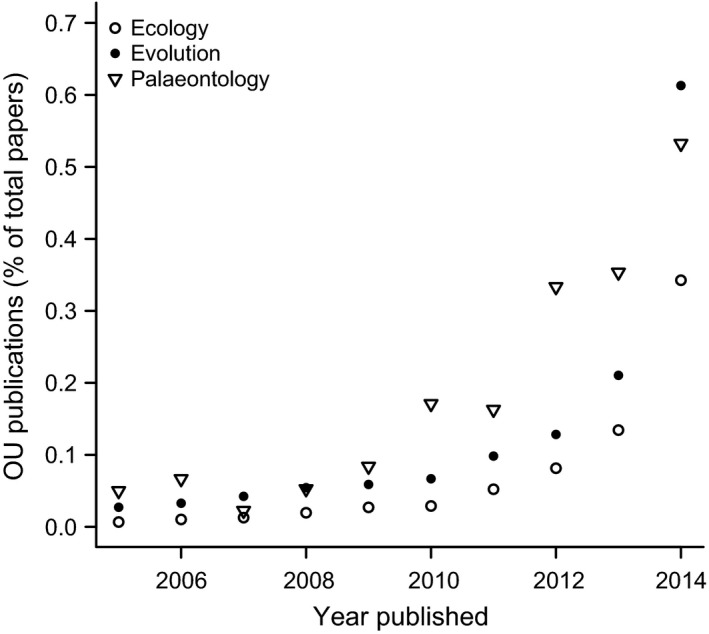
The number of ecology, evolutionary biology and palaeontology papers published between 2005 and 2014 containing the phrase ‘Ornstein Uhlenbeck’, as a proportion of the total number of ecology, evolutionary biology or palaeontology papers published that year. See Supporting Information for details.

The popularity of the OU model has grown extensively in recent years (Fig. 1); even just between 2012 and 2014 over 2500 ecology, evolution and palaeontology papers containing the phrase ‘Ornstein Uhlenbeck’ were published (Google Scholar search 15 March 2015; see Supporting Information). This may partly be because these models are now easy to apply via packages in R (e.g. ouch, GEIGER and OUwie; Butler & King, 2004; Harmon *et al*., 2008; Beaulieu & O'Meara, 2012). Additionally, although the OU model is pattern‐based, it has several attractive biological interpretations. For example, fit to an OU model is used as evidence for processes such as phylogenetic niche conservatism, convergent evolution and stabilizing selection (e.g. Wiens *et al*., 2010; Christin *et al*., 2013; Ingram & Mahler, 2013).

It is important to note, however, that although the OU model is frequently described and interpreted as a model of ‘stabilizing selection’, this is inaccurate and misleading. As formulated by Hansen (1997), a trait has a primary optimum that is the mean of individual species optima for that trait. Under this formulation, α can be considered as the strength of the pull towards a central trait value (the primary optimum; Hansen, 2012). However, this is not an estimate of stabilizing selection in the population genetics sense, where it is a measure of selection within a population towards a fitness optimum on an adaptive landscape (Lande, 1976). This is a qualitatively different process to trait evolution among species which is more akin to a trait tracking movement of the adaptive optima itself.

The OU model is most commonly used to model the evolution of a single continuous character (Table 1; Supporting Information). Usually several models of evolution (Brownian motion, OU, Early Burst, etc.; Harmon *et al*., 2010; Cooper & Purvis, 2010; Cardillo, 2015; Slater, 2015) are fit to the same continuous character and model selection is then used to determine which model best fits the data. OU models can be fit with one optimal trait value, or multiple different optima (Butler & King, 2004; Beaulieu *et al*., 2012). The latter represents evolution under multiple selective regimes, and may be more biologically realistic for many datasets. OU models with various numbers of optima are often included in the pool of evolutionary models being compared (e.g. Christin *et al*., 2013; Table 1). OU models are also commonly used to model phylogenetically structured residual error in evolutionary correlations (Revell, 2010; often colloquially referred to as ‘controlling for phylogeny’; Table 1; Supporting Information).

**Table 1:** The most common uses of Ornstein Uhlenbeck models in ecology, evolutionary biology and palaeontology papers published between 2005 and 2013; see Supporting Information for details

Use of OU model	Optima	No. of papers
Ancestral state reconstruction	Single	8
	Multiple	2
Convergent evolution	Single	0
	Multiple	2
Mode of evolution	Single	31
	Multiple	27
Phylogenetic generalized least squares	Single	35
	Multiple	0
Other	Single	5
	Multiple	5
Total	Single	79
	Multiple	36

As an extension to modelling single traits, phylogenetic generalized least squares (PGLS) models incorporate information about the relationships among species into the error term of a generalized least squares model. This error term generally consists of a variance–covariance matrix of the phylogeny, but various transformations are used (e.g. Pagel's λ; Pagel, 1997) to improve the fit of the model to the data. The α parameter from an OU model can also be used to transform the tree and the variance–covariance matrix. This is rarely interpreted as corresponding to any kind of process; instead it improves the fit of the PGLS models (e.g. Blankers, Adams & Wiens, 2012). However, it is not clear that it was originally intended that the OU model would merely be used in such a context. Finally, the OU model is also used to reconstruct ancestral states (Martins, 1999) and to detect clade‐wide convergent evolution (Ingram & Mahler, 2013; Uyeda & Harmon, 2014).

Most papers use the OU model to model the evolution of a continuous character (Table 1; Supporting Information), so we focus on this use of the OU model in our simulations below. The principles here also apply to a range of other macroevolutionary models that can be fit to continuous data and compared using model testing procedures (e.g. κ, λ, δ, ACDC, Early Burst; Pagel, 1997, 1999; Blomberg, Garland & Ives, 2003; Harmon *et al*., 2008). Note that we focus on OU models with a single stationary optimum trait value because these are more commonly used (Table 1; Supporting Information) and easier to simulate. For discussions on the performance of multiple optima OU models we refer the reader to Beaulieu & O'Meara (2012).

### OU model outline

According to the Brownian model (Cavalli‐Sforza & Edwards, 1967; Felsenstein, 1973), a trait *X* evolves at random at a rate σ:

(1) dX(t)=σdW(t)

where *W*(*t*) is drawn at random from a normal distribution with mean 0 and variance σ^2^. The model assumes that there is no overall drift in the direction of evolution (hence the expectation of *W*(*t*) is zero) and that the rate of evolution is constant. Because the direction of change in trait values at each step is random, Brownian motion is often described as a ‘random walk’. The model assumes the correlation structure among trait values is proportional to the extent of shared ancestry for pairs of species. This means that close relatives will be more similar in their trait values than more distant relatives. It also means that variance in the trait will increase (linearly) in proportion to time. The model has two parameters, the Brownian rate parameter, σ^2^, and the state of the root at time zero, *X*(0).

The OU model (Hansen, 1997; Butler & King, 2004) is a random walk in which trait values revert back towards some ‘optimal’ value, μ (also called θ), with an attraction strength proportional to the parameter α. The model has the following form:

(2) dX(t)=−α(X(t)−μ)+σdW(t)

Note that this model has two parameters in addition to those of the Brownian model: α and μ. α is the strength of evolutionary force that returns traits back towards the long‐term mean, μ, if they evolve away from it. α is sometimes referred to as the ‘rubber band’ parameter because of the way it pulls traits back towards μ. The parameter μ is a long‐term mean, and it is assumed that species traits evolve around this value. For more details see the Appendix.

In many implementations of the OU model (e.g. GEIGER; Harmon *et al*., 2008), μ is the same as the state of the root at time zero, *X*(0). This is referred to as a ‘single stationary peak’ or SSP model (*sensu* Harmon *et al*., 2010). Some implementations also allow users to estimate *X*(0) (e.g. OUwie; Beaulieu & O'Meara, 2012). Where *X*(0) ≠ μ this is referred to as a single peak OU process. However, estimating *X*(0) can sometimes lead to nonsensical values of μ, i.e. far outside the range of values for the trait, so results should be interpreted with caution. *X*(0) can also be defined *a priori*, but this is only appropriate when fossil data, or other independent evidence, allow confident estimates of the root state.

When α is close to zero, evolution is approximately Brownian (but note that in the special case of a single peak OU model with *X*(0) ≠ μ, when μ is zero, evolution approximates Brownian motion with a trend; Hansen, 1997; Benson *et al*., 2014), then as α gets larger the non‐Brownian behaviour of the model starts to become apparent. Eventually, when α is really large, all imprint of history is lost and the trait evolution is essentially a rapid burst at the present. Note that α scales with tree height (i.e. the maximum distance from the root of the tree to the tips); taller trees will have lower α values, all else being equal, because there is more time for traits to return to the optimum value, and thus the strength of the pull towards the optimum, α, can be smaller. Thus, α values need to be interpreted relative to tree height. Generally the simplest solution is to rescale tree heights to 1 (e.g. Ives & Garland, 2010; see simulations below). Ives & Garland (2010) suggest interpreting log (α) after rescaling trees to a height of 1, rather than raw α. They equate –log (α) = 4 as a very low, almost Brownian, value and –log (α) = −4 as a very high value. Others (e.g. Hansen & Bartoszek, 2012; Slater, 2015) prefer to use the phylogenetic half‐life: $t_{\frac{1}{2}}$ (see ‘Recommendations for interpreting α’ below).

## Performance of the OU Model

To explore some issues with the OU model in more detail, we ran a number of simulations designed to mirror the use of OU models in the literature.

### Simulating phylogenies and data

We simulated phylogenies with 25, 50, 100, 150, 200, 500, or 1000 tips under pure birth, constant‐rate Birth–Death (extinction fractions of 0.25, 0.5 and 0.75) or temporally varying speciation rate (speciation rate modelled as time from the root raised to the power 0.2, 0.5, 2 and 5) models. We simulated 1000 phylogenies for each combination of tips and models resulting in 56 000 simulated phylogenies in total. Trees were simulated using the R package TESS (Hohna, 2013). We then simulated the evolution of a single trait under a Brownian motion model on each phylogeny using the R package MOTMOT (Thomas & Freckleton, 2011). All our simulated trees and data are available on GitHub: https://github.com/nhcooper123/OhYou.

### Performance of α and Likelihood ratio tests

To determine whether α is biased under conditions where it should be zero, and whether Likelihood ratio tests are appropriate for use with the OU model, we estimated α for each of our simulated phylogenies and data, and compared the fit of a Brownian model with that of an OU model using a Likelihood ratio test with 1 degree of freedom with the transformPhylo.ML function in MOTMOT (Thomas & Freckleton, 2011; https://github.com/ghthomas/motmot). This mirrors the common situation where researchers fit Brownian and OU models and then use Likelihood ratio tests to select the ‘best’ model. We then estimated the rejection rate of the null (Brownian) model for each set of simulations. We refer to this measure as the Type I error rate for the OU model.

We find that Type I error rates are unacceptably high when tree size is small (Tables 2, 3), i.e. the OU model is often favoured, even though the Brownian model was used to generate the data. For some tree shapes, particularly where speciation rates accelerate towards the present, Type I error remains > 0.05 even for trees with 1000 tips (Table 3). This shows that, in general, analyses based on small datasets are prone to biases that decrease only slowly as the size of the dataset increases. Unfortunately, OU models are often fitted to phylogenies with fewer than 100 taxa (mean = 166.97 ± 43.86, median = 58; Fig. 3; see Supporting Information). We provide recommendations related to these findings below.

**Table 2:** Rejection rate and α estimates for data simulated under a constant‐rate Brownian model on a range of constant‐rate birth death trees

Tree type	Tree size	Rejection rate	Median α (95% quantiles)
d/b = 0	25	0.095	0.165 (0–1.498)
d/b = 0	50	0.074	0.077 (0–0.589)
d/b = 0	100	0.078	0.045 (0–0.343)
d/b = 0	150	0.057	0.034 (0–0.249)
d/b = 0	200	0.055	0.021 (0–0.199)
d/b = 0	500	0.045	0.012 (0–0.115)
d/b = 0	1000	0.039	0.006 (0–0.075)
d/b = 0.25	25	0.093	0.136 (0–0.968)
d/b = 0.25	50	0.092	0.069 (0–0.478)
d/b = 0.25	100	0.065	0.04 (0–0.267)
d/b = 0.25	150	0.065	0.031 (0–0.213)
d/b = 0.25	200	0.054	0.025 (0–0.166)
d/b = 0.25	500	0.047	0.01 (0–0.095)
d/b = 0.25	1000	0.044	0.005 (0–0.06)
d/b = 0.5	25	0.102	0.104 (0–0.851)
d/b = 0.5	50	0.09	0.057 (0–0.394)
d/b = 0.5	100	0.075	0.039 (0–0.219)
d/b = 0.5	150	0.056	0.022 (0–0.154)
d/b = 0.5	200	0.066	0.017 (0–0.138)
d/b = 0.5	500	0.047	0.009 (0–0.07)
d/b = 0.5	1000	0.045	0.004 (0–0.047)
d/b = 0.75	25	0.111	0.068 (0–0.572)
d/b = 0.75	50	0.099	0.044 (0–0.28)
d/b = 0.75	100	0.081	0.022 (0–0.146)
d/b = 0.75	150	0.086	0.019 (0–0.108)
d/b = 0.75	200	0.069	0.012 (0–0.088)
d/b = 0.75	500	0.05	0.006 (0–0.047)
d/b = 0.75	1000	0.045	0.003 (0–0.03)

Tree type refers to the extinction fraction for the birth–death trees. The rejection rate is the proportion of Ornstein Uhlenbeck models favoured relative to a Brownian motion model.

**Table 3:** Rejection rate and α estimates for data simulated under a constant‐rate Brownian model on trees simulated under time‐variable speciation rates

Tree type	Tree size	Rejection rate	Median α (95% quantiles)
Slow speed‐up	25	0.126	0.443 (0–3.18)
Slow speed‐up	50	0.118	0.34 (0–1.963)
Slow speed‐up	100	0.105	0.22 (0–1.324)
Slow speed‐up	150	0.098	0.189 (0–1.113)
Slow speed‐up	200	0.085	0.162 (0–0.9)
Slow speed‐up	500	0.061	0.096 (0–0.545)
Slow speed‐up	1000	0.061	0.065 (0–0.415)
Rapid speed‐up	25	0.191	0.882 (0–7.012)
Rapid speed‐up	50	0.136	0.603 (0–4.082)
Rapid speed‐up	100	0.122	0.527 (0–3.024)
Rapid speed‐up	150	0.122	0.442 (0–2.485)
Rapid speed‐up	200	0.086	0.349 (0–2.01)
Rapid speed‐up	500	0.079	0.241 (0–1.437)
Rapid speed‐up	1000	0.069	0.183 (0–1.083)
Slow slow‐down	25	0.112	0.278 (0–1.792)
Slow slow‐down	50	0.082	0.14 (0–0.985)
Slow slow‐down	100	0.073	0.091 (0–0.549)
Slow slow‐down	150	0.053	0.055 (0–0.388)
Slow slow‐down	200	0.064	0.05 (0–0.349)
Slow slow‐down	500	0.05	0.028 (0–0.209)
Slow slow‐down	1000	0.042	0.017 (0–0.146)
Rapid slow‐down	25	0.093	0.192 (0–1.45)
Rapid slow‐down	50	0.077	0.118 (0–0.854)
Rapid slow‐down	100	0.058	0.061 (0–0.408)
Rapid slow‐down	150	0.064	0.038 (0–0.329)
Rapid slow‐down	200	0.051	0.029 (0–0.278)
Rapid slow‐down	500	0.036	0.014 (0–0.147)
Rapid slow‐down	1000	0.054	0.006 (0–0.11)

The rejection rate is the proportion of Ornstein Uhlenbeck models favoured relative to a Brownian motion model.

**Figure 2:**
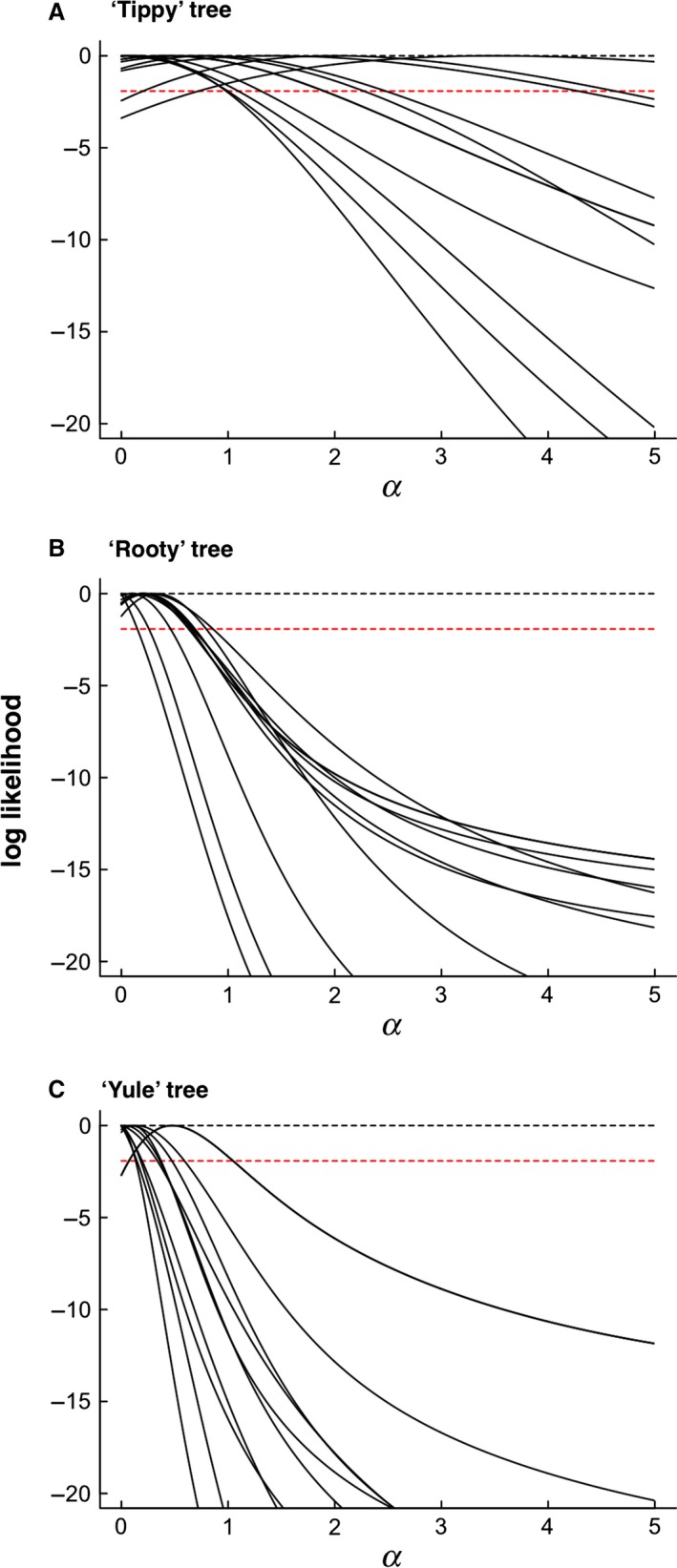
Examples of profile likelihoods for selected simulated datasets for tippy (A), rooty (B) and Yule (C) simulated trees of 50 taxa. Each solid black line represents one simulated dataset selected at random. Tippy trees are those with branching events distributed disproportionately late in the clade's history (i.e. nearer to the present). Rooty trees are those with branching events distributed disproportionately early in the clade's history (i.e. nearer to the root). In all cases the ‘true’ value of α is 0 (black dashed line). The red dashed line represents −1.92 log‐likelihood units from the maximum: using log‐Likelihood ratio tests, values of α yielding values higher than this would be considered statistically indistinguishable from the Maximum Likelihood value.

For small trees the confidence limits on parameter estimates are broad (Fig. 2). However, the median is typically low irrespective of tree size (see Tables 2, 3) and even when the OU model is favoured, scrutiny of the model parameters suggest that the favoured OU model is biologically indistinguishable from Brownian motion. Indeed, examination of the effect of α on the expected covariances among taxa (see Fig. 4) confirms that covariances are barely distinguishable when α < 1 for unit height trees. This suggests that, notwithstanding the elevated Type I errors of OU models, their interpretation requires examination of parameters. We return to the issue of parameter interpretation below, specifically with reference to the increasingly commonly used phylogenetic half‐life.

### Effects of measurement error on OU model performance

Measurement error can strongly affect the results of comparative analyses (Silvestro *et al*., 2015), and appears to influence whether OU is the favoured model across a range of datasets (see Pennell *et al*., 2015). Therefore, we also investigated whether adding error to our simulated data influenced estimates of α.

We used the same procedure as above to simulate trait data under a Brownian motion model with known error. Specifically, we simulated trees under a Yule model with 25, 50, 100, 150, 200, 500 or 1000 tips and added branch length of (1) 1%, (2) 5% or (3) 10% of the tree height to the tips of the simulated trees. We then simulated data under a Brownian model on each tree. All our simulated trees and data are available on GitHub: https://github.com/nhcooper123/OhYou.

We compared the fit of a Brownian model with that of an OU model using Likelihood ratio tests as described above, using the original trees without the addition of extra branch length to the tip edges. Our expectation is that the OU model should fit the data better than the Brownian model because the α parameter should account for much of the error. In this case, rejection of the Brownian model does not represent Type I error because it would be quite correct to reject the Brownian model. However, the reason for the better fit would be entirely unrelated to any macroevolutionary process.

Table 4 shows the proportion of data sets in which the OU model is favoured over the Brownian model for data simulated under Brownian motion with error. The expectation is that the OU model should fit better because the branch length transformation partially captures the non‐Brownian component (the error). There are two points worth noting. First, the frequency with which the OU model is favoured increases with tree size. With as little as 5% error, the OU model becomes extremely difficult to reject, even for trees with just 100 species. This is important for the interpretation of the OU model; we cannot conclude anything about the evolutionary process from a single optimum OU model unless error is adequately accounted for. Second, for moderate amounts of error (5–10%), estimates of α are consistently > 1. Large values of α are similarly difficult to interpret because they are indicative that the signal of the past has been overwritten.

**Table 4:** Rejection rate and α estimates for data simulated under a constant rate Brownian model with 0, 1, 5 or 10% measurement error (m.e.)

Tree size	Rejection rate				Median α (95% quantiles)			
	0%	1%	5%	10%	0%	1%	5%	10%
25	0.095	0.157	0.318	0.478	0.165 (0–1.498)	0.234 (0–1.507)	0.372 (0–5.51)	0.574 (0–20)
50	0.074	0.203	0.542	0.756	0.077 (0–0.589)	0.163 (0–0.789)	0.372 (0–2.2)	0.538 (0.062–14.381)
100	0.078	0.251	0.807	0.957	0.045 (0–0.343)	0.135 (0–0.503)	0.357 (0.06–1.236)	0.54 (0.153–4.094)
150	0.057	0.387	0.947	0.997	0.034 (0–0.249)	0.14 (0–0.445)	0.37 (0.121–1.104)	0.566 (0.224–7.381)
200	0.055	0.487	0.982	1	0.021 (0–0.199)	0.136 (0–0.411)	0.385 (0.142–1.089)	0.544 (0.257–2.98)
500	0.045	0.848	1	1	0.012 (0–0.115)	0.152 (0.035–0.344)	0.394 (0.219–0.919)	0.58 (0.319–3.729)
1000	0.039	0.995	1	1	0.006 (0–0.075)	0.168 (0.079–0.335)	0.417 (0.259–0.844)	0.596 (0.361–2.328)

The rejection rate is the proportion of Ornstein Uhlenbeck models favoured relative to a Brownian motion model.

### Limitations of the OU Model

**Figure 3:**
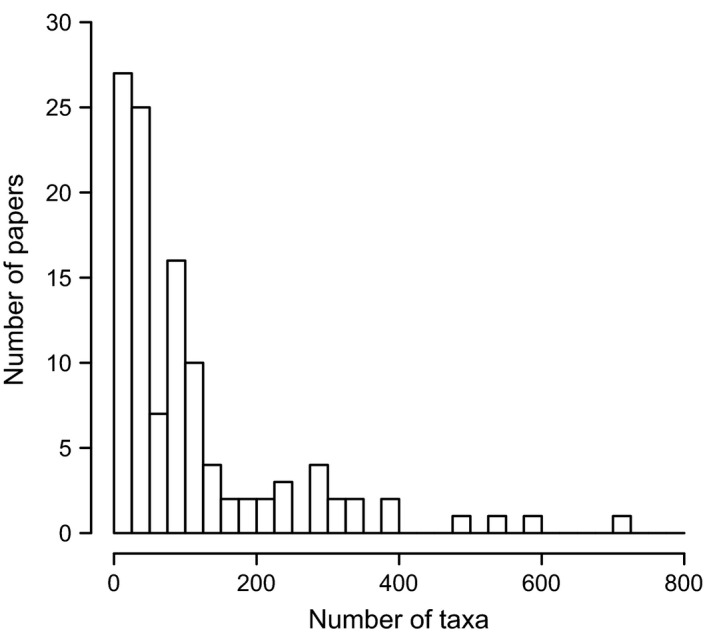
The number of taxa in phylogenies used to fit Ornstein Uhlenbeck models in ecology, evolutionary biology and palaeontology papers published between 2005 and 2014. Two studies with > 3000 taxa have been omitted for clarity. See Supporting Information for details.

Although it is possible to create and implement new models for comparative data that encompass a range of processes, we have to be aware that such models are statistically complex and may behave in unexpected ways. Transformations of the variance–covariance matrix in the Brownian model (e.g. λ; Pagel, 1997) are an attractive and computationally simple way to modify the basic model to include evolutionary processes. But, as first pointed out by Grafen (1989), the statistical consequences of these modifications can include biases and problems with interpretation. The results of our simulations illustrate that such problems can occur under conditions that closely match the size and type of datasets that are commonly used (Fig. 3, Supporting Information, Table S1).

In the case of the OU model, there are several limitations worth highlighting:

1. *Type I error rates are high when sample size is low*. The results of the simulations indicate that, in general, analyses based on small datasets are prone to biases that decrease only slowly as the size of the dataset increases.
2. *Likelihood ratio tests are untrustworthy*. All Likelihood ratio tests assume that the Likelihood ratio statistic is asymptotically (i.e. as sample sizes become large) χ^2^‐distributed. In the OU model α is bounded (i.e. it cannot be any smaller than zero) and has a non‐linear effect on the expected variances, so the assumptions of the Likelihood ratio test are not likely to be upheld for small samples. Our simulations indicate that Likelihood ratio tests should not be relied upon for analyses with small sample sizes, and that for robust inference and testing, alternatives, such as simulation and Monte Carlo Markov chains (MCMC), should be considered [see Recommendations and potential solutions (1) and (2) below]. Note that these issues will also apply when using the Akaike information criterion (AIC) to compare models, as this is essentially the same as performing a Likelihood ratio test. AIC also presents other difficulties when used for complex models such as those used in phylogenetic comparative analyses, so we recommend avoiding them for these reasons.
3. *Measurement error increases Type I error rates*. Our results show that a simple Brownian process can be mistaken for an OU process when a small amount of error is added to the data. The effects of measurement error become more severe with increasing tree size. These limitations mean that when evidence for the OU model is found, the results should be interpreted with caution, particularly where there is likely to be intraspecific variation or measurement error in the data.

## Recommendations and potential solutions

We have highlighted several issues with the OU model above. These can be broadly divided into two classes: (1) fitting the model and (2) interpreting model parameters. These issues can be at least partially addressed with additional or alternative analytical approaches. Below we make recommendations for best practice in fitting the model and approaches to interpret model parameters. We highlight areas of future research that may be important in alleviating outstanding issues.

### Recommendations for model fitting

1. *Simulate under the null model*. OU models should not be applied to small trees. Our simulations indicate that trees with > 200 tips are necessary to obtain acceptable Type I error rates. However, we are cautious about recommending a minimum tree size because the performance of the OU model may vary among datasets for reasons other than tree size. We note also that large trees are particularly susceptible to issues arising from unaccounted measurement error in the data. Instead, we suggest using simulations to assess the model fit. Simulating data under the Brownian model will generate null distributions (e.g. Boettiger *et al*., 2012) and allow straightforward assessment of model fit, for example by generating appropriate critical values for Likelihood ratio tests. Boettiger *et al*. (2012) discuss this at length and other papers use similar approaches (e.g. Martins & Garland, 1991; Freckleton, Harvey & Pagel, 2002).
2. *Consider Bayesian approaches*. An alternative but less explored approach is to use Bayesian methods. Bayesian model fitting has not been widely available for OU model fitting until recently but is now possible in R packages including diversitree (FitzJohn, 2012) and GEIGER (the Single Stationary Peak model in fitContinuousMCMC; Harmon *et al*., 2008) and stand‐alone software including BayesTraits (Pagel & Meade, 2013).

We explored Bayesian methods as a possible remedy to the limitations of fitting OU models in a Likelihood framework. We repeated our simulations in a Bayesian framework implemented in BayesTraits (Pagel & Meade, 2013). We determined the fit to an OU model using Bayes factors estimated from a stepping stone sampling procedure (Xie *et al*., 2010). The marginal likelihoods of the models were calculated using a stepping stone sampler in which 50 stones were drawn from a beta distribution with α = 0.4 and β = 1. Each stone was sampled for 20 000 iterations (with the first 5000 iterations discarded). We use stepping stone sampling as it has been shown to estimate the marginal likelihood better than the harmonic mean (Baele *et al*., 2012). We treated Bayes factors > 2 as evidence favouring the OU model (Kass & Raftery, 1995). We ran the MCMCs for 1 × 10^6^ iterations, disregarding the first 1 × 10^4^ as burn‐in. Following burn‐in the chains were sampled every 1000 iterations to ensure independence of each consecutive sample. Multiple independent chains were run for each analysis to ensure convergence was reached. An important challenge for Bayesian approaches is selection of appropriate priors. We used three alternative sets of priors on α: (1) an exponential distribution with mean = 1, (2) an exponential distribution with mean = 10 and (3) a uniform distribution bounded at 0 and 20. For all analyses we used a uniform −100 to 100 prior for μ and uniform 0–100 prior for σ^2^.

Table 5 shows the results from the exponential prior with mean = 10. This is a broad, liberal prior, but our results are similar regardless of priors. The Bayesian approach results in highly conservative rejection rates regardless of tree shape in the absence of measurement error. While this is encouraging from the perspective of falsely rejecting the Brownian null model, it is also indicative of potentially low statistical power, although testing would be required to confirm this. The Bayesian approach also appears to more readily handle low levels of measurement error (Table 6). With 1% measurement error the Bayesian approach retains acceptable rejection rates (< 0.05) for trees of up to 150 tips. However, more error or larger trees result in the frequent rejection of the Brownian model. As noted above, this is not an issue of Type I error and it is entirely correct that the Brownian model is rejected. In such cases emphasis should shift to how the α parameter is interpreted. Bayesian analysis is a promising approach for OU model fitting. However, further testing of the Bayesian approach to simulated data sets under a wide range of values of α is necessary to fully characterize performance.

**Table 5:** Rejection rate and α estimates for data simulated under a constant‐rate Brownian model on a range of constant‐rate birth death trees using Bayesian methods

Tree type	Tree size	Rejection rate	Median alpha (95% quantiles)
b/d = 0	25	0.021	0.132 (0.025–1.876)
b/d = 0	50	0.003	0.063 (0.017–0.494)
b/d = 0	100	0.001	0.041 (0.012–0.304)
b/d = 0	150	0.001	0.033 (0.009–0.227)
b/d = 0	200	0.002	0.026 (0.008–0.186)
b/d = 0	500	0	0.016 (0.005–0.105)
b/d = 0	1000	0	0.011 (0.004–0.072)
b/d = 0.25	25	0.009	0.1 (0.021–1.189)
b/d = 0.25	50	0.001	0.051 (0.013–0.42)
b/d = 0.25	100	0.002	0.035 (0.01–0.235)
b/d = 0.25	150	0	0.028 (0.007–0.194)
b/d = 0.25	200	0.001	0.024 (0.007–0.149)
b/d = 0.25	500	0	0.013 (0.004–0.093)
b/d = 0.25	1000	0	0.009 (0.003–0.057)
b/d = 0.5	25	0.009	0.077 (0.015–0.922)
b/d = 0.5	50	0.002	0.041 (0.01–0.334)
b/d = 0.5	100	0	0.029 (0.007–0.184)
b/d = 0.5	150	0	0.02 (0.005–0.138)
b/d = 0.5	200	0	0.016 (0.005–0.122)
b/d = 0.5	500	0	0.011 (0.003–0.068)
b/d = 0.5	1000	0	0.007 (0.002–0.046)
b/d = 0.75	25	0.008	0.047 (0.009–0.683)
b/d = 0.75	50	0	0.027 (0.006–0.236)
b/d = 0.75	100	0	0.016 (0.004–0.127)
b/d = 0.75	150	0	0.014 (0.004–0.095)
b/d = 0.75	200	0.001	0.011 (0.003–0.084)
b/d = 0.75	500	0	0.007 (0.002–0.044)
b/d = 0.75	1000	0	0.004 (0.001–0.028)

Tree type refers to the extinction fraction for the birth–death trees. The value of α is the median across simulated data sets based on modal estimates from the posterior distribution. The rejection rate is the proportion of Ornstein Uhlenbeck models favoured relative to a Brownian motion model based on Bayes factors > 2.

**Table 6:** Rejection rate and α estimates for data simulated under a constant rate Brownian model with 0, 1, 5, or 10% measurement error (m.e.) using Bayesian methods

Tree size	Rejection rate				Median α (95% quantiles)			
	0%	1%	5%	10%	0%	1%	5%	10%
25	0.021	0.031	0.109	0.191	0.132 (0.025–1.876)	0.158 (0.03–2.076)	0.286 (0.034–3.726)	0.509 (0.049–5.848)
50	0.003	0.018	0.158	0.332	0.063 (0.017–0.494)	0.107 (0.019–0.723)	0.315 (0.034–2.245)	0.481 (0.057–5.679)
100	0.001	0.033	0.364	0.708	0.041 (0.012–0.304)	0.11 (0.016–0.485)	0.333 (0.044–1.202)	0.521 (0.135–3.183)
150	0.001	0.045	0.637	0.906	0.033 (0.009–0.227)	0.123 (0.016–0.424)	0.357 (0.108–1.074)	0.553 (0.208–4.977)
200	0.002	0.097	0.819	0.978	0.026 (0.008–0.186)	0.126 (0.015–0.399)	0.372 (0.134–1.098)	0.538 (0.243–2.845)
500	0	0.426	1	1	0.016 (0.005–0.105)	0.147 (0.032–0.34)	0.391 (0.214–0.923)	0.575 (0.311–3.36)
1000	0	0.86	1	1	0.011 (0.004–0.072)	0.165 (0.077–0.335)	0.414 (0.256–0.85)	0.592 (0.359–2.278)

The value of α is the median across simulated data sets based on modal estimates from the posterior distribution. The rejection rate is the proportion of Ornstein Uhlenbeck models favoured relative to a Brownian motion model.

### Recommendations for interpreting α

**Figure 4:**
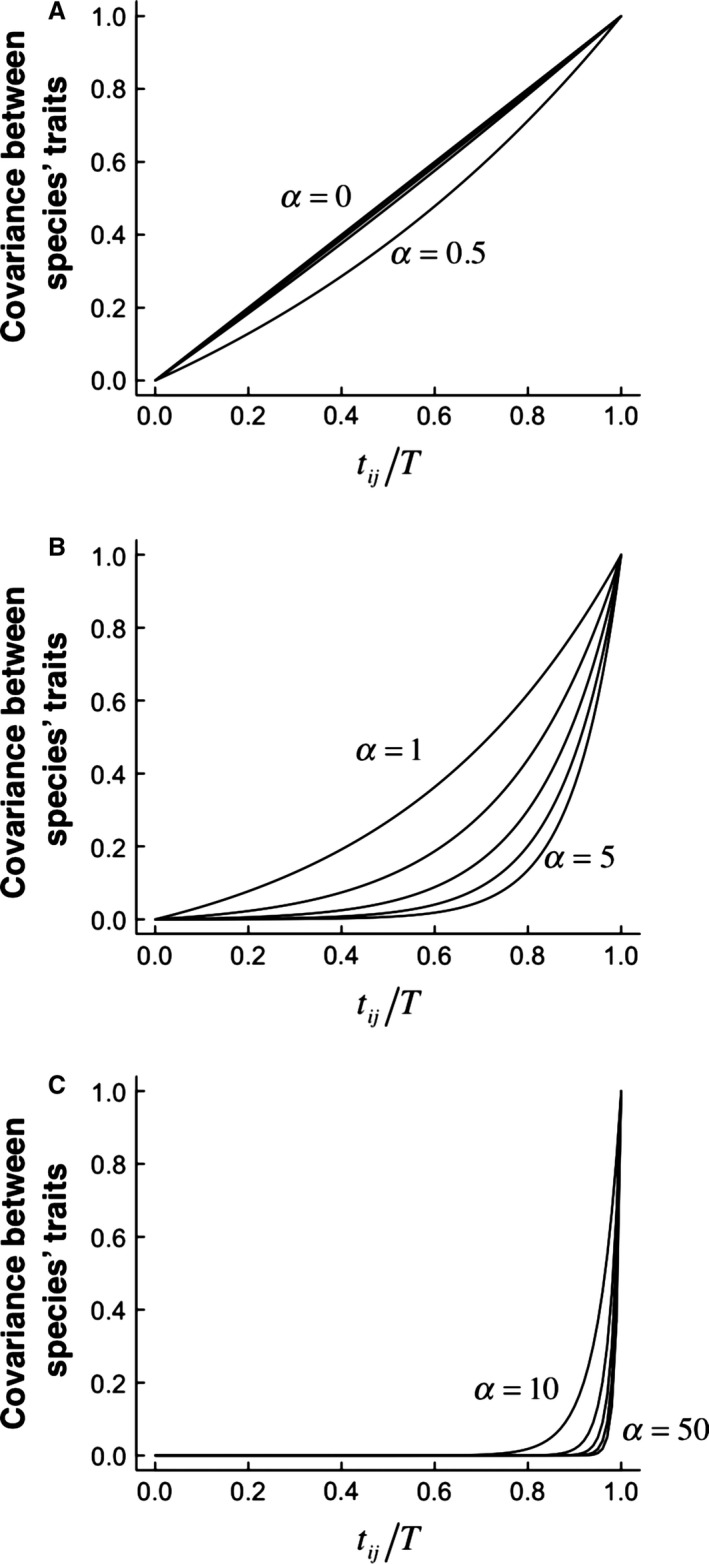
Scaling of expected trait similarity with time since evolutionary divergence predicted by the Ornstein Uhlenbeck model. The covariance between species’ trait values is scaled by the intra‐specific trait variance (i.e. equal to correlation between species’ traits). This is plotted against the relative time of shared history (time at which species branched from each other, divided by the total tree height: *t*_*ij*_/*T*). Different panels show different ranges of ?: (A) α = 0 to 0.5; (B) α = 1 to 5; and (C) α = 10‐50. In (A) trait evolution is essentially Brownian; in (C) it is independent of phylogeny.

1. *Consider plausible alternative hypotheses*. Because the OU model was proposed with evolutionary processes in mind, alternative and arguably more parsimonious explanations for favouring OU models and interpreting non‐zero α are often overlooked. The effects of measurement error in particular suggest that α must always be interpreted with caution. Many issues of misinterpretation can be solved by carefully inspecting the α parameter when an OU model is favoured. Often, when Likelihood ratio tests suggest the OU model should be favoured over the Brownian model, estimates of α are actually very small and biologically indistinguishable from Brownian (e.g. examples in Harmon *et al*., 2010). In these circumstances, it is likely that measurement error, intraspecific variation or phylogenetic uncertainty are generating noise that is more effectively modelled by the extra parameters in the OU model than by the Brownian model alone. Thus, this does not reflect any kind of OU process underlying the data. The similarity between Brownian and OU models with small α is demonstrated in Fig. 4. At the other extreme, as values of α become larger, the effects of changing α on model predictions are increasingly small and large values of α are indistinguishable from white‐noise.

One good strategy for data exploration would be to simulate data under Brownian and the favoured OU model to generate distributions of parameters under known values. These can then be compared with results for your dataset (see Slater, 2014; Slater & Pennell, 2014; for a related approach). This is important because we have shown that the shape of a phylogeny has consequences for parameter biases and hypothesis tests. Any given tree will therefore generate unique parameter estimates. Generating data under the favoured OU model will allow an assessment of whether it is possible to retrieve known values, or whether there is evidence of bias.

*Calculate the phylogenetic half‐life*. The α parameter ranges from zero to infinity (although in practice an upper bound is often set; for example, in GEIGER this is α* = *150; Harmon *et al*., 2008), thus recognizing ‘small’ or ‘large’ values may not be intuitive. α can sometimes be interpreted more easily by using it to estimate the ‘phylogenetic half‐life’ ($t_{\frac{1}{2}}$) of a trait, i.e. the time it takes for a species entering a new niche to evolve halfway toward its new expected optimum (Hansen, 1997), as follows:
  $$t_{\frac{1}{2}}=\frac{ln(2)}{\alpha }$$

If $t_{\frac{1}{2}}$ is short relative to the branch lengths of the phylogeny, evolution towards the optimum trait value is fast, residual phylogenetic correlations are weak and there is little influence of the past on trait values (Hansen, 1997). $t_{\frac{1}{2}}$ equal to the height of the phylogeny is a moderate value (Hansen & Bartoszek, 2012). We would not advise interpreting $t_{\frac{1}{2}}$ as literally being ‘the time it takes for a species entering a new niche to evolve halfway toward its new expected optimum’ (Hansen, 1997). However, if $t_{\frac{1}{2}}$ is extremely large relative to tree height, it suggests that if an OU process is acting, it is extremely weak (e.g. for a clade that is 50 Myr old, a $t_{\frac{1}{2}}$ of 100 Myr suggests a species will not approach the optimum within the temporal range of the clade), and thus should not be interpreted as evidence of any kind of process. As a further note of caution, it is important to recognize that biases in the estimation of α would lead to similar biases in $t_{\frac{1}{2}}$.

*If possible, include ancillary data*. If data on fossils are available then these could be incorporated into the analysis (Slater, Harmon & Alfaro, 2012). Indeed, if fossil taxa can be reliably placed in the phylogeny then they may improve model accuracy and a strong case can be made that fossils should be included. On the other hand, if there is substantial uncertainty in fossil placement then they should be treated cautiously. Placement of fossil taxa is implemented in GEIGER (Harmon *et al*., 2008) and BayesTraits (Pagel & Meade, 2013). A caution here is that the OU model for non‐ultrametric trees has to be carefully parameterized because for non‐ultrametric trees the co‐variances depend on both the shared distances between species and the distance of a node to the nearest tip (see eqn A6). This creates potential problems in parameterization and in interpretation because the variance–covariance matrix is no longer tree‐like; for example, related species can effectively become more similar to one another than to themselves – an inherently non tree‐like pattern (Slater, 2014). Some current implementations of the OU model are based on transforming the tree directly, rather than transforming the variance–covariance matrix (e.g. MOTMOT; Thomas & Freckleton, 2011). These implementations should not be used with fossil data.

## Conclusions and Outstanding Issues

A recurring theme from our simulations is that interpretation of OU models is not straightforward. More focus is needed on the interpretation of α rather than simply model fit. Even when the OU model is favoured, α may be so small as to be indistinguishable from Brownian motion in any biological sense. It would clearly be very useful to have estimates of measurement error for all species traits, although the inclusion of species‐specific variances has to be done carefully (e.g. Grafen, 1989). Several approaches for accounting for error have been proposed and warrant wider implementation for OU models and other models of trait evolution (e.g. Lynch, 1991; Martins & Hansen, 1997; Ives, Midford & Garland, 2007; Hansen & Bartoszek, 2012; Rohlfs, Harrigan & Nielsen, 2014), but it is outside our scope to explore these here.

Indeed, the problems that we report are not limited to OU models. Any model of trait evolution that attempts to account for non‐Brownian components of trait variation is susceptible to being misled by measurement error, and in some scenarios measurement error can also incorrectly favour Brownian motion over the true model, e.g. if the true model is Early Burst. The fundamental problem is that rejection of the Brownian model in favour of another model does not necessarily say anything about process. This problem can be alleviated to some extent if model comparisons are set in a firm hypothesis‐testing framework in which alternative hypotheses make clear predictions of emerging patterns that can be unambiguously associated with particular models (e.g. Cooper, Freckleton & Jetz, 2011), although this does not appear to be possible for comparisons of the single stationary peak OU model with noisy Brownian processes. We should therefore not use any statistical model without thinking carefully about the limits in terms of both data and interpretation.

## Epilogue: The Challenges of Open and Reproducible Science

At the symposium that generated this special issue, one of us (N.C.) gave a talk on Open Science and reproducibility. We have therefore tried to make this paper as open and reproducible as possible. All simulated phylogenies, data and R code are available on GitHub: https://github.com/nhcooper123/OhYou. However, although our R code is provided, it is disorganized and thus difficult to use. We also do not provide an automated way of reproducing the data collection for our literature review. Nor do we use tools such as Travis CI (travis‐ci.org), Docker (www.docker.com) or packrat (Ushey *et al*., 2015) to increase the reproducibility of our analyses. Additionally, we use BayesTraits to run our Bayesian analyses. BayesTraits is free to download as a binary executable for various platforms but it is not Open Source. This means that while BayesTraits analyses are reproducible, the software has limitations with respect to long‐term development of the code. We included this epilogue to highlight that fully reproducible and Open Science is challenging, but we are trying to improve. With a little effort most people should be able to produce something vaguely reproducible, and to provide their data and code, moving us all slightly closer to truly reproducible and Open Science.

## Supporting information


**Table S1.** Details of the papers used in our literature review.

## Appendix

According to the Brownian model, a trait *X* evolves at random at a rate σ:

(A1) dX(t)=σdW(t)

where *W*(*t*) is a white‐noise function and is a random variate drawn from a normal distribution with mean 0 and variance σ^2^. This model assumes that there is no overall drift in the direction of evolution [hence the expectation of *W*(*t*) is zero] and that the rate of evolution is constant. The model has two parameters, σ and the state of the root at time zero, *X* (0). The Brownian model predicts after a time *T* the variance in trait value *X*_*i*_ for species *i* is:

(A2) $$var\left(X_{i}\right)=\sigma^{2}T$$

and the covariance in traits for species *i* and *j* is:

(A3) $$cov\left(X_{i},X_{j}\right)=\sigma^{2}t_{ij}$$

where *t*_*ij*_ is the shared evolutionary pathway for species *i* and *j*, i.e. the time at which they last shared a common ancestor. Equations A2 and A3 encapsulate the simplicity of the Brownian model, namely it predicts that variances accrue as a linear function of time.The OU model describes a mean‐reverting process and has the following form, adding an extra term to the Brownian model:

(A4) dX(t)=−α(X(t)−μ)+σdW(t)

The parameter μ is a long‐term mean, and it is assumed that species evolve around this value. σ is the strength of evolutionary force that returns traits back towards the mean if they evolve away. This model has two parameters in addition to those of the Brownian model, α and μ. The OU model predicts that after a time *T* for a species *i*, the variance in trait value *X*_*i*_ is:

(A5) $$var\left(X_{i}\right)=\frac{\sigma^{2}}{2\alpha }1-e^{-2\alpha T}$$

And for a pair of species *i* and *j*, the covariance in traits is:

(A6) $$cov\left(X_{i},X_{j}\right)=\frac{\sigma^{2}}{2\alpha }e^{-2\alpha (T-t_{ij})}\left(1-e^{-2\alpha t_{ij}}\right)$$

The variances and covariances predicted by eqns A5 and A6 are more complex than those predicted by the Brownian model. In the light of the results above, some properties of this model are worth highlighting:

1. If α is small then evolution is approximately Brownian: if α is small then % 1 ‐ *e* ^‐2α*T*^ ≈ 2*αT*, i.e. traits accrue variance as if evolving according to a Brownian process.
2. If species *i* and *j* diverged recently, evolution is approximately Brownian: if two species diverged recently, then *T* ‐ *t*_*ij*_ ≈ 0 and hence $cov\left(X_{i},X_{j}\right)\approx 1-e^{-2\alpha t_{ij}}\approx 2\alpha t_{ij}$. Thus, recently diverged species provide little information relevant to estimating non‐Brownian evolution according to an OU process.

In the long term, the imprint of history is weakened: if *T* is large (i.e. evolution proceeds for a long time), eqn A5 predicts that the variance in *X*_*i*_ tends to a constant, i.e. because the expected value of *X*_*i*_ is μ. Similarly, in eqn A4, the covariance between traits tends to a constant because *T* becomes large relative to *t*_*ij*_. Consequently for large groups the model implies that the imprint of history is weak.
