# Supplementary material for: A cautionary note on the use of Ornstein Uhlenbeck models in macroevolutionary studies
Source: Biol J Linn Soc Lond. 2015 Dec 1;118(1):64–77. doi: 10.1111/bij.12701 (PMC4949538; doi:10.1111/bij.12701)
Supplement: Supplementary file 1 — Table S1. Details of the papers used in our literature review. [file BIJ-118-64-s001.pdf]

Supporting Information

Literature review methods

To get an overview of the use of OU models in ecology, evolution and palaeontology, we used Google Scholar (accessed 13th March 2015) to locate papers published between 2005 (when the R package ouch was released; Butler and King 2004) and 2014 that contained the terms "Ornstein Uhlenbeck" and either "'ecology", or "evolution" and "biology" (the "biology" term was added to omit physics papers which also use the term "evolution"), or "paleo/palaeo". We also recorded the total number of papers containing the terms "ecology", or "evolution" and "biology", or "paleo/palaeo", published between 2005 and 2014 and plotted the number of OU papers published each year as a proportion of the total number of papers published (Fig. 1 in the main text).

Next we filtered our Google Scholar search results to focus on empirical papers using OU models (rather than pure methods papers) published in the following journals: The American Naturalist, Ecology Letters, Evolution, Journal of Evolutionary Biology, Nature, Proceedings of the National Academy of Sciences, USA, Proceedings of the Royal Society B: Biological Sciences, and Science. We only include papers up to the end of 2013 to ensure completeness.

For each of these papers we recorded the number of species in the analysis, the study group (amphibians, birds, fish, mammals, reptiles, invertebrates or plants), the statistical package or specific R package used to fit the models, and the reason the authors state for using an OU model (ancestral state reconstructions, detecting convergent evolution, controlling for phylogeny, selecting a model of trait evolution, or other). Where papers included multiple analyses using different numbers of species we used the median number of taxa. Where papers had multiple study

groups, statistics/R packages or reasons for fitting OU models we counted them in each relevant category. We summarise these results in Figures 1 and 2 and Table 1 in the main text, and the full dataset is available in Table S1 along with the full list of references.

Literature review results

In total, our literature search found 3720 papers published between 2005 and 2014, and the number has increased substantially since 2005 (Fig. 1 in main text). Most papers fit OU models to phylogenies with fewer than 100 taxa (mean = 166.97 $\pm$ 43.86, median = 58, Figure 2 in main text and Table S1). The majority of papers fit OU models using R packages, particularly GEIGER and, although other uses are becoming more common, most papers use OU models in an effort to discern the "best" model of trait evolution or to control for phylogenetic non-independence (Table S1).

Table S1: Details of the papers used in our literature review. For each paper we recorded the study taxon, the number of tips in the phylogeny used to fit the Ornstein Uhlenbeck (OU) model, how the authors used the OU model in the paper, and the statistical package (usually R) used to carry out the analyses. For a full reference list see below. Journal abbreviations: Am Nat = The American Naturalist, ELE = Ecology Letters, JEB = Journal of Evolutionary Biology, PRSB = Proceedings of the Royal Society B: Biological Sciences, PNAS = Proceedings of the National Academy of the USA. Number of tips in the phylogeny the OU model was fitted to. Where there were multiple analyses in a paper we use the median number of tips. All packages mentioned are R packages apart from BayesTraits, COMPARE, MATLAB, Mesquite, PAM, PDAP and PDTREE.

**Paper**	**Year**	**Journal**	**Taxon**	**Ntips**	**Use in paper**	**Stats/R package**
Hansen and Orzack 2005	2005	Evolution	insects	15	other	OUCH precursor?
Edwards and Donoghue 2006	2006	Am Nat	plants	12	ancestral state reconstruction	COMPARE
Gvoždík and Damme 2006	2006	Evolution	amphibians	10	phylogenetic correction	COMPARE
Halsey et al. 2006	2006	Am Nat	birds/mammals	90	phylogenetic correction	Custom code
Ives and Godfray 2006	2006	Am Nat	insects	8	phylogenetic signal	MATLAB
Valiente-Banuet et al. 2006	2006	PNAS	plants	47	model of evolution	? OUCH
Clabaut et al. 2007	2007	Evolution	fish	45	phylogenetic correction	APE
Gomez and Théry 2007	2007	Am Nat	birds	40	model of evolution	OUCH
Hipp 2007	2007	Evolution	plants	53	model of evolution	BayesTraits
Rezende et al. 2007	2007	Nature	plants/insects	?	phylogenetic signal	? OUCH

Spoor et al. 2007	2007	PNAS	mammals	210	phylogenetic correction	PDAP
StuartFox et al. 2007	2007	Am Nat	reptiles	21	phylogenetic correction	COMPARE
Buchwalter et al. 2008	2008	PNAS	insects	21	phylogenetic correction	MATLAB
Dumont and Payseur 2008	2008	Evolution	mammals	13	model of evolution	OUCH
Hansen et al. 2008	2008	Evolution	mammals	105	other	SLOUCH
Smith et al. 2008	2008	Evolution	plants	15	phylogenetic correction	APE
Warne and Charnov 2008	2008	Am Nat	reptiles	71	phylogenetic correction	MATLAB
Adams et al. 2009	2009	PRSB	amphibians	10	model of evolution	OUCH
Addison et al. 2009	2009	PRSB	birds	23	phylogenetic correction	MATLAB
Agrawal et al. 2009	2009	PNAS	plants	53	model of evolution	GEIGER
Bergmann et al. 2009	2009	Evolution	reptiles	38	model of evolution	OUCH
Collar et al. 2009	2009	Evolution	fish	29	model of evolution	OUCH
Gonzalez-Voyer et al. 2009	2009	PRSB	fish	39	phylogenetic correction	APE/COMPARE
Goodman et al. 2009	2009	Evolution	reptiles	20	phylogenetic correction	COMPARE
Huey et al. 2009	2009	PRSB	reptiles	70	phylogenetic correction	MATLAB
Kozak et al. 2009	2009	Evolution	amphibians	184	model of evolution	GEIGER
Labra et al. 2009	2009	Am Nat	reptiles	83	other	SLOUCH
Rezende et al. 2009	2009	ELE	fish	116	model of evolution	PDTREE
Swanson and Garland 2009	2009	Evolution	birds	44	phylogenetic correction	MATLAB

Van Buskirk 2009	2009	JEB	amphibians	82	other	SLOUCH
Burbrink and Pyron 2010	2010	Evolution	reptiles	29	model of evolution	GEIGER
Cooper and Purvis 2010	2010	Am Nat	mammals	45	model of evolution	GEIGER
Edwards and Smith 2010	2010	PNAS	plants	300	model of evolution	OUCH
Harmon et al. 2010	2010	Evolution	multiple	17	model of evolution	GEIGER
Helmus et al. 2010	2010	ELE	zooplankton	15	phylogenetic correction	?
Kalinka et al. 2010	2010	Nature	insects	6	model of evolution	OUCH
Kozak and Wiens 2010b	2010	Am Nat	amphibians	84	model of evolution/ ancestral state reconstruction	GEIGER/ OUCH
Kozak and Wiens 2010a	2010	ELE	amphibians	11	model of evolution	OUCH
Ord et al. 2010	2010	Evolution	reptiles	16	other	SLOUCH
Price et al. 2010	2010	Evolution	fish	122	model of evolution	GEIGER
Slater et al. 2010	2010	PRSB	mammals	84	model of evolution	OUCH
Angielczyk et al. 2011	2011	Evolution	reptiles	8	model of evolution	OUCH
Benesh et al. 2011	2011	Evolution	helminths	310	model of evolution	OUCH
Collar et al. 2011	2011	Evolution	reptiles	37	model of evolution	Brownie
Derryberry et al. 2011	2011	Evolution	birds	285	model of evolution	GEIGER
Galvan and Moller 2011	2011	JEB	birds	323	phylogenetic correction	COMPARE
Gonzalez-Voyer and Kolm 2011	2011	JEB	fish	49	model of evolution	GEIGER

Ord et al. 2011	2011	Evolution	multiple	23	phylogenetic signal	SLOUCH
Oufiero et al. 2011	2011	Evolution	reptiles	106	phylogenetic correction	SLOUCH
Perez et al. 2011	2011	JEB	mammals	29	phylogenetic correction	APE
Raia and Meiri 2011	2011	Evolution	mammals	842	model of evolution	MOTMOT
Rosas-Guerrero et al. 2011	2011	Evolution	plants	20	phylogenetic correction	APE
Setiadi et al. 2011	2011	Am Nat	amphibians	22	model of evolution	OUCH
Smith et al. 2011	2011	Evolution	reptiles	15	model of evolution	GEIGER
Tulli et al. 2011	2011	JEB	reptiles	29	phylogenetic correction	?
Turbill et al. 2011	2011	PRSB	mammals	19	phylogenetic correction	GEIGER
Valido et al. 2011	2011	JEB	plants	111	phylogenetic correction	APE
Wiens et al. 2011	2011	ELE	amphibians	337	model of evolution/ phylogenetic correction	GEIGER/ COMPARE
Weir and Wheatcroft 2011	2011	PRSB	birds	232	model of evolution	GEIGER
Beaulieu et al. 2012	2012	Evolution	plants	590	model of evolution	OUwie
Betancur-R et al. 2012	2012	ELE	fish	123	model of evolution	GEIGER
Blankers et al. 2012	2012	JEB	amphibians	189	phylogenetic correction	GEIGER
Boettiger et al. 2012	2012	Evolution	reptiles	23	ancestral state reconstruction	OUCH
Burbrink et al. 2012	2012	PRSB	multiple	41	model of evolution	Custom code
Calosi et al. 2012	2012	JEB	insects	25	phylogenetic correction	MATLAB

Claramunt et al. 2012a	2012	Am Nat	birds	290	model of evolution	GEIGER
Claramunt et al. 2012b	2012	PRSB	birds	282	model of evolution	GEIGER
Davis et al. 2012	2012	JEB	insects	53	phylogenetic correction	SLOUCH
Diniz-Filho et al. 2012	2012	Evolution	mammals	209	other	PAM
Fusco et al. 2012	2012	Evolution	trilobites	60	model of evolution	?
Gomez-Mestre et al. 2012	2012	Evolution	amphibians	720	phylogenetic correction	APE
Ingram et al. 2012	2012	JEB	food webs	20	model of evolution	GEIGER
Kellermann et al. 2012a	2012	Evolution	insects	94	phylogenetic correction	SLOUCH
Kellermann et al. 2012b	2012	PNAS	insects	94	phylogenetic correction	SLOUCH
Nogueira et al. 2012	2012	JEB	plants	105	phylogenetic signal	GEIGER
Ord 2012	2012	JEB	reptiles	32	ancestral state reconstruction	SLOUCH
Pearse and Hipp 2012	2012	Evolution	plants	56	phylogenetic correction	SLOUCH
Pellissier et al. 2012	2012	JEB	insects	83	model of evolution	? APE
Price et al. 2012	2012	Evolution	fish	50	model of evolution	GEIGER
Sallan and Friedman 2012	2012	PRSB	fish	100	model of evolution	GEIGER
Santana et al. 2012	2012	Evolution	mammals	85	model of evolution	OUCH
Schmerler et al. 2012	2012	PRSB	plants	88	phylogenetic correction	nlme
Smith 2012	2012	Evolution	birds	42	phylogenetic correction	APE/nlme
Sookias et al. 2012	2012	PRSB	multiple	43	model of evolution	GEIGER

Stireman et al. 2012	2012	JEB	insects	24	model of evolution	GEIGER
Voje and Hansen 2012	2012	Evolution	insects	30	phylogenetic correction	SLOUCH
Weir et al. 2012	2012	Evolution	birds	232	model of evolution	GEIGER
Arbour and López-Fernández 2013	2013	PRSB	fish	27	model of evolution	OUCH
Benesh et al. 2013	2013	Am Nat	helminths	143	phylogenetic correction	APE
Blackburn et al. 2013	2013	Evolution	amphibians	18	model of evolution	GEIGER
Christin et al. 2013	2013	PNAS	plants	545	model of evolution	GEIGER/OUCH
Frédérich et al. 2013	2013	Am Nat	fish	208	model of evolution	OUwie
Friedman et al. 2013	2013	Evolution	birds	15	model of evolution	OUCH
Guerrero et al. 2013	2013	PNAS	plants/reptiles	49	ancestral state reconstruction	GEIGER/ COMPARE
Hertz et al. 2013	2013	Evolution	reptiles	100	model of evolution	GEIGER
Hossie et al. 2013	2013	JEB	amphibians/ reptiles	104	phylogenetic correction	GEIGER
Knope and Scales 2013	2013	JEB	fish	26	model of evolution	OUCH
Kostikova et al. 2013	2013	Am Nat	plants	68	model of evolution	OUwie
Lambert and Wiens 2013	2013	Evolution	reptiles	117	ancestral state reconstruction	GEIGER
Lapiedra et al. 2013	2013	PRSB	birds	154	model of evolution	OUwie

Litsios et al. 2013	2013	Evolution	plants	382	model of evolution	OUwie
López-Fernández et al. 2013	2013	Evolution	fish	135	model of evolution	GEIGER
Machac et al. 2013	2013	Evolution	mammals	231	model of evolution	GEIGER
Mahler et al. 2013	2013	Science	reptiles	100	convergent evolution	SURFACE
Maia et al. 2013	2013	PNAS	birds	47	model of evolution	OUwie
Mirceta et al. 2013	2013	Science	mammals	130	phylogenetic correction/ ancestral state reconstruction	Mesquite/ MATLAB
Moen et al. 2013	2013	PRSB	amphibians	44	convergent evolution	GEIGER
Pérez i de Lanuza et al. 2013	2013	JEB	reptiles	42	ancestral state reconstruction	GEIGER
Pienaar et al. 2013	2013	ELE	birds	382	model of evolution	SLOUCH
Quintero and Wiens 2013	2013	ELE	multiple	500	ancestral state reconstruction	GEIGER/COMPARE
Ryan and Shaw 2013	2013	PRSB	mammals	34	phylogenetic correction	MATLAB
Seddon et al. 2013	2013	PRSB	birds	153	model of evolution	? GEIGER
Tanabe and Sota 2013	2013	Evolution	millipedes	84	phylogenetic correction	APE
Voje et al. 2013	2013	JEB	fish	87	other	SLOUCH
Wiens et al. 2013	2013	Evolution	reptiles	117	ancestral state reconstruction	GEIGER

Literature review references

Adams, D. C., C. M. Berns, K. H. Kozak, and J. J. Wiens. 2009. Are rates of species diversification correlated with rates of morphological evolution? Proceedings of the Royal Society B: Biological Sciences 276:2729-2738.

Addison, B., K. C. Klasing, W. D. Robinson, S. H. Austin, and R. E. Ricklefs. 2009. Ecological and life-history factors influencing the evolution of maternal antibody allocation: a phylogenetic comparison. Proceedings of the Royal Society B: Biological Sciences 276:3979-3987.

Agrawal, A. A., M. Fishbein, R. Halitschke, A. P. Hastings, D. L. Rabosky, and S. Rasmann. 2009. Evidence for adaptive radiation from a phylogenetic study of plant defenses. Proceedings of the National Academy of Sciences 106:18067-18072.

Angielczyk, K. D., C. R. Feldman, and G. R. Miller. 2011. Adaptive evolution of plastron shape in emydine turtles. Evolution 65:377-394.

Arbour, J. H., and H. López-Fernández. 2013. Ecological variation in South American geophagine cichlids arose during an early burst of adaptive morphological and functional evolution. Proceedings of the Royal Society B: Biological Sciences 280.

Beaulieu, J. M., D.-C. Jhwueng, C. Boettiger, and B. C. OMeara. 2012. Modeling stabilizing selection: expanding the Ornstein-Uhlenbeck model of adaptive evolution. Evolution 66:2369-2383.

Benesh, D. P., J. C. Chubb, and G. A. Parker. 2011. Exploitation of the same trophic link favors convergence of larval life-history strategies in complex life cycle helminths. Evolution 65:2286-2299.

Benesh, D. P., J. C. Chubb, and G. A. Parker. 2013. Complex life cycles: why refrain from growth before reproduction in the adult niche? The American Naturalist 181:39-51.

Bergmann, P. J., J. J. Meyers, and D. J. Irschick. 2009. Directional evolution of

stockiness coevolves with ecology and locomotion in lizards. Evolution 63:215-227.

Betancur-R, R., G. Ort, A. M. Stein, A. P. Marceniuk, and R. Alexander Pyron. 2012. Apparent signal of competition limiting diversification after ecological transitions from marine to freshwater habitats. Ecology Letters 15:822-830.

Blackburn, D. C., C. D. Siler, A. C. Diesmos, J. A. McGuire, D. C. Cannatella, and R. M. Brown. 2013. An adaptive radiation of frogs in a Southeat Asian island archipelago. Evolution 67:26312646.

Blankers, T., D. C. Adams, and J. J. Wiens. 2012. Ecological radiation with limited morphological diversification in salamanders. Journal of evolutionary biology 25:634-646.

Boettiger, C., G. Coop, and P. Ralph. 2012. Is your phylogeny informative? Measuring the power of comparative methods. Evolution 66:2240-2251.

Buchwalter, D. B., D. J. Cain, C. A. Martin, L. Xie, S. N. Luoma, and T. Garland. 2008. Aquatic insect ecophysiological traits reveal phylogenetically based differences in dissolved cadmium susceptibility. Proceedings of the National Academy of Sciences 105:8321-8326.

Burbrink, F. T., X. Chen, E. A. Myers, M. C. Brandley, and R. A. Pyron. 2012. Evidence for determinism in species diversification and contingency in phenotypic evolution during adaptive radiation. Proceedings of the Royal Society B: Biological Sciences 279:4817-4826.

Burbrink, F. T., and R. A. Pyron. 2010. How does ecological opportunity influence rates of speciation, extinction, and morphological diversification in New World ratsnakes (tribe Lampropeltini)? Evolution 64:934-943.

Calosi, P., D. T. Bilton, J. I. Spicer, W. C. E. P. Verberk, A. Atfield, and T. Garland. 2012. The comparative biology of diving in two genera of European Dytiscidae (Coleoptera). Journal of Evolutionary Biology 25:329-341.

Christin, P.-A., C. P. Osborne, D. S. Chatelet, J. T. Columbus, G. Besnard, T. R. Hodkinson, L. M. Garrison, M. S. Vorontsova, and E. J. Edwards. 2013. Anatomical

enablers and the evolution of C4 photosynthesis in grasses. Proceedings of the National Academy of Sciences 110:1381-1386.

Clabaut, C., P. M. E. Bunje, W. Salzburger, and A. Meyer. 2007. Geometric morphometric analyses provide evidence for the adaptive character of the Tanganyikan cichlid fish radiations. Evolution 61:560-578.

Claramunt, S., E. P. Derryberry, R. T. Brumfield, and J. V. Remsen Jr. 2012a. Ecological opportunity and diversification in a continental radiation of birds: climbing adaptations and cladogenesis in the Furnariidae. The American Naturalist 179:649-666.

Claramunt, S., E. P. Derryberry, J. V. Remsen, and R. T. Brumfield. 2012b. High dispersal ability inhibits speciation in a continental radiation of passerine birds. Proceedings of the Royal Society B: Biological Sciences 279:1567-1574.

Collar, D. C., B. C. O'Meara, P. C. Wainwright, and T. J. Near. 2009. Piscivory limits diversification of feeding morphology in centrarchid fishes. Evolution 63:1557-1573.

Collar, D. C., J. A. Schulte Ii, and J. B. Losos. 2011. Evolution of extreme body size disparity in monitor lizards (Varanus). Evolution 65:2664-2680.

Cooper, N., and A. Purvis. 2010. Body size evolution in mammals: complexity in tempo and mode. The American Naturalist 175:727-738.

Davis, R. B., J. Javois, J. Pienaar, E. unap, and T. Tammaru. 2012. Disentangling determinants of egg size in the Geometridae (Lepidoptera) using an advanced phylogenetic comparative method. Journal of Evolutionary Biology 25:210-219.

Derryberry, E. P., S. Claramunt, G. Derryberry, R. T. Chesser, J. Cracraft, A. Aleixo, J. Prez-Emn, J. J. V. Remsen, and R. T. Brumfield. 2011. Lineage diversification and morphological evolution in a large-scale continental radiation: the Neotropical ovenbirds and woodcreepers (Aves: Furnariidae). Evolution 65:2973-2986.

Diniz-Filho, J. A. F., T. F. Rangel, T. Santos, and L. Mauricio Bini. 2012.

Exploring patterns of interspecific variation in quantitative traits using sequential phylogenetic eigenvector regressions. Evolution 66:1079-1090.

Dumont, B. L., and B. A. Payseur. 2008. Evolution of the genomic rate of recombination in mammals Evolution 62:276-294.

Edwards, E. J., and M. J. Donoghue. 2006. Pereskia and the origin of the cactus life-form. American Naturalist 167:777-793.

Edwards, E. J., and S. A. Smith. 2010. Phylogenetic analyses reveal the shady history of C4 grasses. Proceedings of the National Academy of Sciences 107:2532-2537.

Frédérich, B., L. Sorenson, F. Santini, G. J. Slater, and M. E. Alfaro. 2013. Iterative ecological radiation and convergence during the evolutionary history of damselfishes (Pomacentridae). The American Naturalist 181:94-113.

Friedman, N. R., K. J. McGraw, and K. E. Omland. 2013. Evolution of carotenoid pigmentation in caciques and meadowlarks (Icteridae): repeated gains of red plumage coloration by carotenoid C4-oxygenation. Evolution in press.

Fusco, G., J. T. Garland, G. Hunt, and N. C. Hughes. 2012. Developmental trait evolution in trilobites. Evolution 66:314-329.

Galvan, I., and A. P. Moller. 2011. Brain size and the expression of pheomelanin-based colour in birds. Journal of Evolutionary Biology 24:999-1006.

Gomez, D., and M. Théry. 2007. Simultaneous crypsis and conspicuousness in color patterns: comparative analysis of a neotropical rainforest bird community. The American Naturalist 169:S42-S61.

Gomez-Mestre, I., R. A. Pyron, and J. J. Wiens. 2012. Phylogenetic analyses reveal unexpected patterns in the evolution of reproductive modes in frogs. Evolution 66:3687-3700.

Gonzalez-Voyer, A., and N. Kolm. 2011. Rates of phenotypic evolution of ecological characters and sexual traits during the Tanganyikan cichlid adaptive radiation. Journal of Evolutionary Biology 24:2378-2388.

Gonzalez-Voyer, A., S. Winberg, and N. Kolm. 2009. Social fishes and single mothers: brain evolution in African cichlids. Proceedings of the Royal Society B: Biological Sciences 276:161-167.

Goodman, B. A., S. C. Hudson, J. L. Isaac, and L. Schwarzkopf. 2009. The evolution of body shape in response to habitat: is reproductive output reduced in flat lizards? Evolution 63:1279-1291.

Guerrero, P. C., M. Rosas, M. T. K. Arroyo, and J. J. Wiens. 2013. Evolutionary lag times and recent origin of the biota of an ancient desert (AtacamaSechura). Proceedings of the National Academy of Sciences 110:11469-11474.

Gvoždík, L., and R. V. Damme. 2006. Triturus newts defy the running-swimming dilemma. Evolution 60:2110-2121.

Halsey, L. G., P. J. Butler, and T. M. Blackburn. 2006. A phylogenetic analysis of the allometry of diving. The American Naturalist 167:276-287.

Hansen, T. F., and S. H. Orzack. 2005. Assessing current adaptation and phylogenetic inertia as explanations of trait evolution: the need for controlled comparisons. Evolution 59:2063-2072.

Hansen, T. F., J. Pienaar, and S. H. Orzack. 2008. A comparative method for studying adaptation to a randomly evolving environment. Evolution 62:1965-1977.

Harmon, L. J., J. B. Losos, T. Jonathan Davies, R. G. Gillespie, J. L. Gittleman, W. Bryan Jennings, K. H. Kozak, M. A. McPeek, F. Moreno Roark, and T. J. Near. 2010. Early bursts of body size and shape evolution are rare in comparative data. Evolution 64:2385-2396.

Helmus, M. R., W. Keller, M. J. Paterson, N. D. Yan, C. H. Cannon, and J. A. Rusak. 2010. Communities contain closely related species during ecosystem disturbance. Ecology Letters 13:162-174.

Hertz, P. E., Y. Arima, A. Harrison, R. B. Huey, J. B. Losos, and R. E. Glor. 2013. Asynchronous evolution of physiology and morphology in Anolis lizards. Evolution 67:2101-2113.

Hipp, A. L. 2007. Nonuniform processes of chromosome evolution in sedges (Carex: Cyperaceae). Evolution 61:2175-2194.

Hossie, T. J., C. Hassall, W. Knee, and T. N. Sherratt. 2013. Species with a chemical defence, but not chemical offence, live longer. Journal of Evolutionary Biology 26:1598-1602.

Huey, R. B., C. A. Deutsch, J. J. Tewksbury, L. J. Vitt, P. E. Hertz, H. J. Álvarez Pérez, and T. Garland. 2009. Why tropical forest lizards are vulnerable to climate warming. Proceedings of the Royal Society of London Series B-Biological Sciences 276:1939-1948.

Ingram, T., L. J. Harmon, and J. B. Shurin. 2012. When should we expect early bursts of trait evolution in comparative data? Predictions from an evolutionary food web model. Journal of Evolutionary Biology 25:1902-1910.

Ives, A. R., and H. C. J. Godfray. 2006. Phylogenetic analysis of trophic associations. The American Naturalist 168:E1-E14.

Kalinka, A. T., K. M. Varga, D. T. Gerrard, S. Preibisch, D. L. Corcoran, J. Jarrells, U. Ohler, C. M. Bergman, and P. Tomancak. 2010. Gene expression divergence recapitulates the developmental hourglass model. Nature 468:811-814.

Kellermann, V., V. Loeschcke, A. A. Hoffmann, T. N. Kristensen, C. Fljgaard, J. R. David, J.-C. Svenning, and J. Overgaard. 2012a. Phylogenetic constraints in key functional traits behind species' climate niches: patterns of desiccation and cold resistance across 95 Drosophila species. Evolution 66:3377-3389.

Kellermann, V., J. Overgaard, A. A. Hoffmann, C. Fljgaard, J.-C. Svenning, and V. Loeschcke. 2012b. Upper thermal limits of Drosophila are linked to species distributions and strongly constrained phylogenetically. Proceedings of the National Academy of Sciences 109:16228-16233.

Knope, M. L., and J. A. Scales. 2013. Adaptive morphological shifts to novel habitats in marine sculpin fishes. Journal of Evolutionary Biology 26:472-482.

Kostikova, A., G. Litsios, N. Salamin, and P. B. Pearman. 2013. Linking

life-history traits, ecology, and niche breadth evolution in North American eriogonoids (Polygonaceae). The American Naturalist 182:760-774.

Kozak, K. H., R. W. Mendyk, and J. J. Wiens. 2009. Can parallel diversification occur in sympatry? Repeated patterns of body-size evolution in coexisting clades of North American salamanders. Evolution 63:1769-1784.

Kozak, K. H., and J. J. Wiens. 2010a. Accelerated rates of climatic-niche evolution underlie rapid species diversification. Ecology Letters 13:1378-1389.

Kozak, K. H., and J. J. Wiens. 2010b. Niche conservatism drives elevational diversity patterns in Appalachian salamanders. The American Naturalist 176:40-54.

Labra, A., J. Pienaar, and T. F. Hansen. 2009. Evolution of thermal physiology in Liolaemus lizards: adaptation, phylogenetic inertia, and niche tracking. The American Naturalist 174:204-220.

Lambert, S. M., and J. J. Wiens. 2013. Evolution of viviparity: a phylogenetic test of the cold-climate hypothesis in phrynosomatid lizards. Evolution 67:2614-2630.

Lapiedra, O., D. Sol, S. Carranza, and J. M. Beaulieu. 2013. Behavioural changes and the adaptive diversification of pigeons and doves. Proceedings of the Royal Society B: Biological Sciences 280.

Litsios, G., R. O. West, A. Kostikova, F. Forest, C. Lexer, H. P. Linder, P. B. Pearman, N. E. Zimmermann, and N. Salamin. 2013. Effects of a fire response trait on diversification in replicated radiations. Evolution in press.

López-Fernández, H., J. H. Arbour, K. O. Winemiller, and R. L. Honeycutt. 2013. Testing for ancient adaptive radiations in Neotropical cichlid fishes. Evolution 67:1321-1337.

Machac, A., D. Storch, and J. J. Wiens. 2013. Ecological causes of decelerating diversification in carnivoran mammals. Evolution 67:24232433.

Mahler, D. L., T. Ingram, L. J. Revell, and J. B. Losos. 2013. Exceptional convergence on the macroevolutionary landscape in island lizard radiations. Science 341:292-295.

Maia, R., D. R. Rubenstein, and M. D. Shawkey. 2013. Key ornamental innovations facilitate diversification in an avian radiation. Proceedings of the National Academy of Sciences in press.

Mirceta, S., A. V. Signore, J. M. Burns, A. R. Cossins, K. L. Campbell, and M. Berenbrink. 2013. Evolution of mammalian diving capacity traced by myoglobin net surface charge. Science 340:1234192.

Moen, D. S., D. J. Irschick, and J. J. Wiens. 2013. Evolutionary conservatism and convergence both lead to striking similarity in ecology, morphology and performance across continents in frogs. Proceedings of the Royal Society B: Biological Sciences 280:20132156.

Nogueira, A., P. J. Rey, and L. G. Lohmann. 2012. Evolution of extrafloral nectaries: adaptive process and selective regime changes from forest to savanna. Journal of Evolutionary Biology 25:2325-2340.

Ord, T. J. 2012. Historical contingency and behavioural divergence in territorial Anolis lizards. Journal of Evolutionary Biology 25:2047-2055.

Ord, T. J., L. King, and A. R. Young. 2011. Contrasting theory with the empirical data of species recognition. Evolution 65:2572-2591.

Ord, T. J., J. A. Stamps, and J. B. Losos. 2010. Adaptation and plasticity of animal communication in fluctuating environments. Evolution 64:3134-3148.

Oufiero, C. E., G. E. A. Gartner, S. C. Adolph, and T. Garland. 2011. Latitudinal and climatic variation in body size and dorsal scale counts in Sceloporus lizards: a phylogenetic perspective. Evolution 65:3590-3607.

Pearse, I. S., and A. L. Hipp. 2012. Global patterns of leaf defenses in oak species. Evolution 66:2272-2286.

Pellissier, L., S. Rasmann, G. Litsios, K. Fiedler, A. Dubuis, J. Pottier, and A. Guisan. 2012. High host-plant nitrogen content: a prerequisite for the evolution of antcaterpillar mutualism? Journal of Evolutionary Biology 25:1658-1666.

Pérez i de Lanuza, G., E. Font, and J. L. Monterde. 2013. Using visual

modelling to study the evolution of lizard coloration: sexual selection drives the evolution of sexual dichromatism in lacertids. Journal of Evolutionary Biology 26:1826-1835.

Perez, S. I., J. Klaczko, G. Rocatti, and S. F. Dos Reis. 2011. Patterns of cranial shape diversification during the phylogenetic branching process of New World monkeys (Primates: Platyrrhini). Journal of Evolutionary Biology 24:1826-1835.

Pienaar, J., A. Ilany, E. Geffen, and Y. Yom-Tov. 2013. Macroevolution of lifehistory traits in passerine birds: adaptation and phylogenetic inertia. Ecology Letters 16:571576.

Price, S. A., J. J. Tavera, T. J. Near, and P. Wainwright. 2012. Elevated rates of morphological and functional diversification in reef-dwelling haemulid fishes. Evolution 67:417428.

Price, S. A., P. C. Wainwright, D. R. Bellwood, E. Kazancioglu, D. C. Collar, and T. J. Near. 2010. Functional innovations and morphological diversification in parrotfish. Evolution 64:30573068.

Quintero, I., and J. J. Wiens. 2013. Rates of projected climate change dramatically exceed past rates of climatic niche evolution among vertebrate species. Ecology letters 16:1095-1103.

Raia, P., and S. Meiri. 2011. The tempo and mode of evolution: body sizes of island mammals. Evolution 65:1927-1934.

Rezende, E. L., E. M. Albert, M. A. Fortuna, and J. Bascompte. 2009. Compartments in a marine food web associated with phylogeny, body mass, and habitat structure. Ecology Letters 12:779-788.

Rezende, E. L., J. E. Lavabre, P. R. Guimares, P. Jordano, and J. Bascompte. 2007. Non-random coextinctions in phylogenetically structured mutualistic networks. Nature 448:925-928.

Rosas-Guerrero, V., M. Quesada, W. S. Armbruster, R. Prez-Barrales, and S. D. Smith. 2011. Influence of pollination specialization and breeding system on floral

integration and phenotypic variation in Ipomoea. Evolution 65:350-364.

Ryan, T. M., and C. N. Shaw. 2013. Trabecular bone microstructure scales allometrically in the primate humerus and femur. Proceedings of the Royal Society B: Biological Sciences 280.

Sallan, L. C., and M. Friedman. 2012. Heads or tails: staged diversification in vertebrate evolutionary radiations. Proceedings of the Royal Society B: Biological Sciences 279:2025-2032.

Santana, S. E., I. R. Grosse, and E. R. Dumont. 2012. Dietary hardness, loading behavior, and the evolution of skull form in bats. Evolution 66:2587-2598.

Schmerler, S. B., W. L. Clement, J. M. Beaulieu, D. S. Chatelet, L. Sack, M. J. Donoghue, and E. J. Edwards. 2012. Evolution of leaf form correlates with tropicaltemperate transitions in Viburnum (Adoxaceae). Proceedings of the Royal Society B: Biological Sciences 279:3905-3913.

Seddon, N., C. A. Botero, J. A. Tobias, P. O. Dunn, H. E. A. MacGregor, D. R. Rubenstein, J. A. C. Uy, J. T. Weir, L. A. Whittingham, and R. J. Safran. 2013. Sexual selection accelerates signal evolution during speciation in birds. Proceedings of the Royal Society B: Biological Sciences 280.

Setiadi, M. I., J. A. McGuire, R. M. Brown, M. Zubairi, D. T. Iskandar, N. Andayani, J. Supriatna, and B. J. Evans. 2011. Adaptive radiation and ecological opportunity in Sulawesi and Philippine fanged frog (Limnonectes) communities. The American Naturalist 178:221-240.

Slater, G. J., S. A. Price, F. Santini, and M. E. Alfaro. 2010. Diversity versus disparity and the radiation of modern cetaceans. Proceedings of the Royal Society B: Biological Sciences 277:3097-3104.

Smith, K. L., L. J. Harmon, L. P. Shoo, and J. Melville. 2011. Evidence of constrained phenotypic evolution in a cryptic species complex of agamid lizards. Evolution 65:976-992.

Smith, N. D. 2012. Body mass and foraging ecology predict evolutionary

patterns of skeletal pneumaticity in the diverse "waterbird" clade. Evolution 66:1059-1078.

Smith, S. D., C. Ané, and D. A. Baum. 2008. The role of pollinator shifts in the floral diversification of Iochroma (Solanaceae). Evolution 62:793-806.

Sookias, R. B., R. J. Butler, and R. B. J. Benson. 2012. Rise of dinosaurs reveals major body-size transitions are driven by passive processes of trait evolution. Proceedings of the Royal Society B: Biological Sciences 279:2180-2187.

Spoor, F., T. Garland, G. Krovitz, T. M. Ryan, M. T. Silcox, and A. Walker. 2007. The primate semicircular canal system and locomotion. Proceedings of the National Academy of Sciences 104:10808-10812.

Stireman, J. O., H. Devlin, and P. Abbot. 2012. Rampant host- and defensive phenotype-associated diversification in a goldenrod gall midge. Journal of Evolutionary Biology 25:1991-2004.

StuartFox, D., A. Moussalli, and M. J. Whiting. 2007. Natural selection on social signals: signal efficacy and the evolution of chameleon display coloration. The American Naturalist 170:916-930.

Swanson, D. L., and J. T. Garland. 2009. The evolution of high summit metabolism and cold tolerance in birds and its impact on present-day distributions. Evolution 63:184-194.

Tanabe, T., and T. Sota. 2013. Both male and female novel traits promote the correlated evolution of genitalia between the sexes in an arthropod. Evolution in press.

Tulli, M. J., V. Abdala, and F. B. Cruz. 2011. Relationships among morphology, clinging performance and habitat use in Liolaemini lizards. Journal of Evolutionary Biology 24:843-855.

Turbill, C., C. Bieber, and T. Ruf. 2011. Hibernation is associated with increased survival and the evolution of slow life histories among mammals. Proceedings of the Royal Society B: Biological Sciences 278:3355-3363.

Valido, A., H. M. Schaefer, and P. Jordano. 2011. Colour, design and reward: phenotypic integration of fleshy fruit displays. Journal of Evolutionary Biology 24:751-760.

Valiente-Banuet, A., A. V. Rumebe, M. Verd, and R. M. Callaway. 2006. Modern Quaternary plant lineages promote diversity through facilitation of ancient Tertiary lineages. Proceedings of the National Academy of Sciences 103:16812-16817.

Van Buskirk, J. 2009. Getting in shape: adaptation and phylogenetic inertia in morphology of Australian anuran larvae. Journal of evolutionary biology 22:1326-1337.

Voje, K. L., and T. F. Hansen. 2012. Evolution of static allometries: adaptive change in allometric slopes of eye span in stalk-eyed flies. Evolution 67:453-467.

Voje, K. L., A. B. Mazzarella, T. F. Hansen, K. øOstbye, T. Klepaker, A. Bass, A. Herland, K. M. Brum, F. Gregersen, and L. A. Vøollestad. 2013. Adaptation and constraint in a stickleback radiation. Journal of evolutionary biology 26:2396-2414.

Warne, R. W., and E. L. Charnov. 2008. Reproductive allometry and the sizenumber tradeoff for lizards. The American Naturalist 172:E80-E98.

Weir, J. T., and D. Wheatcroft. 2011. A latitudinal gradient in rates of evolution of avian syllable diversity and song length. Proceedings of the Royal Society B: Biological Sciences 278:1713-1720.

Weir, J. T., D. J. Wheatcroft, and T. D. Price. 2012. The role of ecological constraint in driving the evolution of avian song frequency across a latitudinal gradient. Evolution 66:2773-2783.

Wiens, J. J., K. H. Kozak, and N. Silva. 2013. Diversity and niche evolution along aridity gradients in North American lizards (Phrynosomatidae). Evolution 67:1715-1728.

Wiens, J. J., R. A. Pyron, and D. S. Moen. 2011. Phylogenetic origins of local-scale diversity patterns and the causes of Amazonian megadiversity. Ecology Letters 14:643-652.
